# Impact of Multimodal Surgical Resection and Personalized Targeted Therapy on Survival Outcomes in Early-Stage Malignant Pleural Mesothelioma: A Meta-Analysis

**DOI:** 10.7759/cureus.84640

**Published:** 2025-05-22

**Authors:** Alexis Quetzalcoatl Vega Morales, Jorge Luis Rivera Gastelum, Alfonso J Massé Ponce, Alexis Agustin A Dunay Silva, Emiliano Banda Dávila, Andrea Virginia Rivera Aguirre, Odalys Brigitte Villares Santillán, Michael David Naranjo Venegas

**Affiliations:** 1 General Surgery, Mexican Social Security Institute Regional General Hospital 180, Jalisco, MEX; 2 General Surgery, Universidad Autónoma de Sinaloa, Culiacán, MEX; 3 General Surgery, Hospital General Tacuba Institute of Security and Social Services for State Workers, Mexico City, MEX; 4 Internal Medicine, Hospital Barros Luco Trudeau, San Miguel, CHL; 5 Medicine, Universidad Autónoma de Zacatecas “Francisco García Salinas”, Zacatecas, MEX; 6 Medicine, Ministry of Public Health, Guayaquil, ECU; 7 Medicine, Universidad Central del Ecuador, Quito, ECU

**Keywords:** extrapleural pneumonectomy, hemithoracic radiotherapy, malignant pleural mesothelioma, multimodal therapy, pleurectomy/decortication, systematic review and meta analysis

## Abstract

Malignant pleural mesothelioma (MPM) is an aggressive malignancy with limited evaluates the efficacy and safety of these treatments. A systematic review and meta-analysis were conducted, including 14 studies that compared multimodal therapies for early-stage MPM. Continuous variables were analyzed using random-effects modeling, with heterogeneity assessed using I² statistics. The primary outcomes included physical function, social function, and lethargy. The meta-analysis found no statistically significant differences between experimental and control groups in terms of physical function (standardized mean difference [SMD]: -0.34, 95% confidence interval [CI]: -1.14 to 0.45), social function (SMD: 0.01, 95% CI: -0.52 to 0.53), or lethargy (SMD: -0.34, 95% CI: -0.96 to 0.27). Heterogeneity across studies was moderate to high (I²: 47%-76%). These findings suggest limited improvements in quality-of-life domains with experimental approaches compared to controls. This systematic review and meta-analysis highlights the need for individualized, multimodal treatment strategies in MPM management. While extrapleural pneumonectomy and extended pleurectomy/decortication offer specific benefits, their impact on quality of life varies and may not consistently provide significant improvements. Future research should focus on large-scale, randomized trials with standardized protocols to optimize treatment outcomes.

## Introduction and background

The rare and extremely aggressive cancer known as malignant pleural mesothelioma (MPM) is mostly caused by asbestos exposure. It may develop due to previous radiation therapy or a genetic predisposition [1,2]. The prognosis is still bleak despite substantial research efforts and systemic therapy advancements. Oncologic surgical resection is impossible because of the tumor's distinctive laminar growth along the visceral and parietal pleura. Multimodal treatment has consequently emerged as the accepted strategy for early-stage MPM to improve postoperative local tumor control [3]. 

Approximately 2,500 new cases of MPM are recorded each year in the United States, whereas over 160,000 new cases of lung cancer are reported yearly [4]. Moreover, a majority of the instances had a history of asbestos exposure. According to population-based studies, the median survival times for patients with MPM under different treatment plans range from eight to 11 months. Current treatment strategies typically involve combinations of surgical resection, chemotherapy (used as neoadjuvant or adjuvant therapy), and radiation. An analysis of 14,228 MPM cases from the Surveillance, Epidemiology, and End Results (SEER) database identified surgical intervention as an independent factor associated with improved survival [5]. A further SEER study, analyzing 5,937 cases from 1990 to 2004, revealed that surgical treatment was performed in 22% of patients [6]. Since Sugarbaker and his team reported favorable outcomes with extrapleural pneumonectomy (EPP) in 1999, the procedure has been adopted by several high-volume thoracic surgery centers for treating early-stage MPM [7]. In the 2000s, there was significant optimism that tumor control could be further enhanced through a maximal radical approach combining surgery with neoadjuvant chemotherapy and adjuvant radiotherapy [8].

Personalized targeted therapies also hold promise for improving survival outcomes in early-stage MPM, a rare and aggressive cancer. While chemotherapy remains the standard of care, studies are exploring how to better target specific genetic and molecular changes in MPM to improve treatment efficacy. Immunotherapy, particularly immune checkpoint inhibitors, has shown encouraging results in some patients, and ongoing research continues to explore the potential of other targeted therapies [9].

The primary objective of this meta-analysis is to evaluate the impact of multimodal surgical resection, including approaches such as extrapleural pneumonectomy (EPP) and extended pleurectomy and decortication (P/D), or with personalized targeted therapies, on survival outcomes in patients with early-stage MPM. The study aims to analyze complications, lethargy, physical function, and social function to determine the effectiveness of these interventions in improving patient prognosis. By synthesizing data from multiple studies, it intends to provide evidence-based recommendations for optimizing treatment strategies for early-stage MPM.

## Review

Methods

Study Design

The current study is a systematic review and meta-analysis of 14 clinical trials, guided by the Preferred Reporting Items for Systematic Reviews and Meta-Analyses (PRISMA) model [10].

Eligibility Criteria

The inclusion and exclusion criteria for this meta-analysis are presented in Table 1.

**Table 1:** Inclusion and exclusion criteria EPP: Extrapleural pneumonectomy; EPD: Extended pleurectomy and decortication; HITOC: Hyperthermic intrathoracic chemotherapy

Category	Inclusion criteria	Exclusion criteria
Patient population	Studies focusing on early-stage malignant pleural mesothelioma (MPM), specifically stages I–III	Studies focusing solely on advanced-stage MPM (stage IV) or other types of mesothelioma, such as peritoneal mesothelioma
Treatment approach	Studies evaluating multimodal treatment, including surgical interventions (EPP or EPD) combined with adjunct therapies (HITOC, neoadjuvant chemotherapy, or targeted therapy)	Articles that do not evaluate multimodal treatment or focus exclusively on single-modality treatments
Outcomes reported	Studies reporting survival outcomes, perioperative morbidity, or quality of life metrics	Studies lacking survival outcomes, perioperative morbidity data, or quality of life metrics
Publication type	Peer-reviewed journal articles	Non-peer-reviewed articles, case reports, letters to the editor, conference abstracts, and review articles without original data
Language	Articles published in English	Studies published in languages other than English
Methodological rigor	Studies providing sufficient methodological details for critical appraisal and data extraction	Articles with insufficient methodological details, such as unclear patient selection criteria or missing outcome data
Study design	Prospective and retrospective cohort studies, as well as randomized controlled trials	Duplicate publications or datasets already included in other eligible studies
Publication period	Articles published within the past two decades to ensure relevance to contemporary treatment practices	Studies conducted more than 20 years ago or using outdated treatment modalities and not relevant to current clinical practices

Search Strategy

The search strategy adopted for this systematic review includes an in-depth search for relevant literature across various databases such as PubMed, Google Scholar, and Cochrane Library. PRISMA guidelines were followed throughout the search for the articles. Different journal titles, abstracts, and full-text articles were found. Boolean operators AND/OR were used for the search strategy on different search engines. Multiple filters were also applied to make the search more specific.

We found a total of 14 studies (n=564) that were eligible as per the inclusion criteria and covered the terms: “malignant pleural mesothelioma,” “MPM,” “early-stage mesothelioma,” “multimodal treatment,” “extrapleural pneumonectomy,” “extended pleurectomy and decortication,” “EPD,” “hyperthermic intrathoracic chemoperfusion,” “HITOC,” “targeted therapy,” “personalized therapy,” “chemotherapy,” “survival outcomes,” “perioperative morbidity,” and “quality of life.”

Selection Process

The screening of the articles was conducted in two stages. The initial stage of screening involved reviewing the titles and abstracts of all articles identiﬁed from the selected electronic databases. A list of papers was drawn up from this review for potential inclusion. In the second stage, the full text of the selected articles was retrieved.

Uniform data extraction tables containing data on ﬁrst author, year of publication, study design, geographical location, country of study, sample size, sampling method, data source, instrument, standard errors and standard deviations, and associated factors, were created for each eligible paper. Any disagreements were resolved by discussion in conjunction with the senior author’s decision. The studies for the meta-analysis were selected based on the similarity of the outcome measures. The studies in which the data was not sufficiently similar or the ones where the methodological quality was poor, were not included.

Data Items

**Figure 1:**
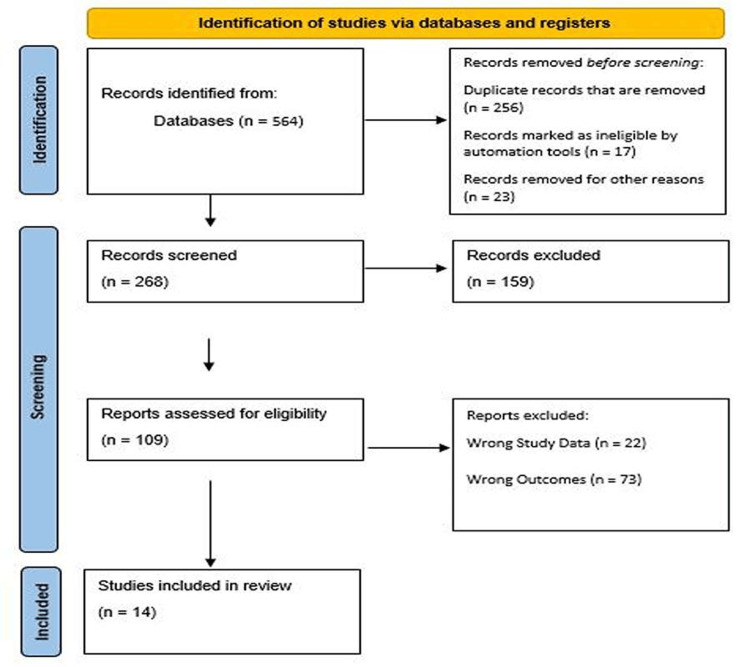
PRISMA flowchart of the included studies PRISMA: Preferred Reporting Items for Systematic Review and Meta-Analysis

The total sample size for the selected literature (n=14) was scrutinized after the secondary screening protocol was completed. We used the PRISMA standards to create a flow diagram (Figure 1) for the selected studies from journals and other independent resources (if the reports were available).

After the study selection process was complete, we tabulated the study interventions one-by-one against the study population and the outcomes studied. Only the relevant themes of the outcome were mentioned in the synthesis table. 

Risk of Bias (RoB) Assessment

We sought digital/online tools for RoB assessment of the studies selected for the meta-analysis. Cochrane RoB version 2 online tool was used to assess 7 domains of risk occurring in the primary studies [11]. The RoB domains that were analyzed for the meta-analysis were as follows: (1) Random sequence generation (selection bias), (2) Allocation concealment (selection bias), (3) blinding of participants and personnel (performance bias), (4) blinding of outcome assessment (detection bias), (5) incomplete outcome data (attrition bias), (6) selective reporting (reporting bias), and (7) other bias. Continuous data was extracted for the statistical analysis from six primary studies. We created a “forest plot” using Review Manager (RevMan version 5.4, Cochrane Collaboration, London, UK) for the meta-analysis. Three researchers collected comparable and pooled the data for the analytical tool. All the data was available in the form of continuous variables. The data for the meta-analysis is provided in the results section.

Statistical Analysis

The meta-analysis was performed using Review Manager (RevMan) software to synthesize both dichotomous and continuous outcomes across studies. Dichotomous outcomes, such as complication and lethargy rates, were analyzed using odds ratios (ORs) with corresponding 95% confidence intervals (CIs), while continuous outcomes, including physical and social function, were assessed using either mean differences (MDs) or standardized mean differences (SMDs), depending on the consistency of measurement scales across studies. Heterogeneity was evaluated using the Chi² test and I² statistic, with I² values >50% considered indicative of substantial heterogeneity. A random-effects model was applied when heterogeneity was moderate to high, while a fixed-effects model was used when heterogeneity was low. Statistical significance was defined as a p-value less than 0.05. Forest plots were generated to visually present pooled estimates, confidence intervals, and heterogeneity levels, enabling clear interpretation of effect sizes and direction across the included studies.

Publication Bias Assessment

Bias in the analysis was minimized by (1) selecting high-quality research and thorough literature review, (2) eliminating the double standard concerning peer review and informed consent applied to clinical research and practice, (3) requiring peer reviewers to acknowledge conflicts of interest, (5) replacing ordinary review articles with meta-analyses. Systematic reviews and narrative reviews were frequently excluded from the literature to maintain the standards of the study. These guidelines detect and remove bias in the study protocol in accordance with Chalmers et al. (1990) stages of removing publication bias [12]. All the studies chosen for the meta- analysis were found to have a “low” overall risk of bias. A “traffic light” figure was plotted using this data for randomization. A summary of the RoB was also mentioned for collaborator convenience.

Results

A PRISMA flowchart was made for the included studies (Figure 1). It is a widely used tool to illustrate the study selection process in systematic reviews and meta-analyses. It visually details each stage, from identification and screening to eligibility and inclusion, enhancing transparency and reproducibility. By documenting the number of studies at each step and the reasons for exclusions, the flowchart provides readers with a clear understanding of the methodology and rigor behind the study selection process. 

Study Characteristics

The final sample for the systematic analysis included 14 peer-reviewed studies. The demographic data and study characteristics of the included trials have been summarized in Table 2.

**Table 2:** Characteristics of all the included studies QOL: Quality of life

Study	Location	Study design	Population	Sample size	Intervention	Follow up	Outcome measures
Cho et al., 2021 [13]	Canada	Single-center, phase 2 trial	Patients aged 18 years or older with histologically proven, resectable, cT1–3N0M0 disease who had previously untreated malignant pleural mesothelioma	96	Extrapleural pneumonectomy	10 years	Complications
Clive et al., 2016 [14]	UK	Multicenter, open-label, phase 3, randomized controlled trial	Patients with histocytologically proven mesothelioma who had undergone large-bore pleural interventions	203	Surgical and large-bore pleural procedures	5 years	Complications, lethargy
de Perrot et al., 2016 [15]	Canada	Clinical trial	Patients with malignant pleural mesothelioma	62	Extrapleural pneumonectomy	-	Complications
Weder et al., 2007 [16]	Switzerland	Clinical trial	Patients with malignant pleural mesothelioma	58	Extrapleural pneumonectomy		QOL, tiredness
Ambrogi et al., 2012 [17]	Italy	Clinical trial	Patients with malignant pleural mesothelioma	29	Extrapleural pneumonectomy	36 months	QOL
Ambrogi et al., 2009 [18]	Italy	Clinical trial	Patients with malignant pleural mesothelioma	16	Extradural pneumonectomy	24 months	QOL
Burkholder et al., 2015 [19]	USA	Clinical trial	Patients with malignant pleural mesothelioma	36	Extrapleural pneumonectomy		QOL and pulmonary function
Mollberg et al., 2012 [20]	USA	Clinical trial	Patients with malignant pleural mesothelioma	28	Radical pleurectomy decortication	9 months	QOL
Ploenes et al., 2012 [21]	Germany	Clinical trial	Patients with malignant pleural mesothelioma	48	Pleurectomy/decortication or extrapleural pleuropneumonectomy		QOL
Tanaka et al., 2016 [22]	Japan	Clinical trial	Patients with malignant pleural mesothelioma	22	Surgical treatment for malignant pleural mesothelioma	12 months	QOL
Stahel et al., 2015 [23]	Switzerland	A randomized, international, multicenter phase 2 trial	Patients with malignant pleural mesothelioma	151	Extrapleural pneumonectomy		Locoregional relapse-free survival, rate of complete macroscopic resection (R0-R1), median survival
Treasure et al., 2011 [24]	UK	Randomised feasibility study	Patients with malignant pleural mesothelioma	112	Extrapleural pneumonectomy		Overall survival (OS), Perioperative mortality, QOL, complications
Sayan et al., 2023 [25]	Turkey	Clinical trial	Patients with malignant pleural mesothelioma	64	Complete multimodal therapy	24 months	Median overall survival (21 months), 5-year survival rate
Klotz et al., 2022 [8]	Germany	Clinical trial	Patients with malignant pleural mesothelioma	182	Multimodal therapy	24 months	Complications

RoB in studies

**Figure 2:**
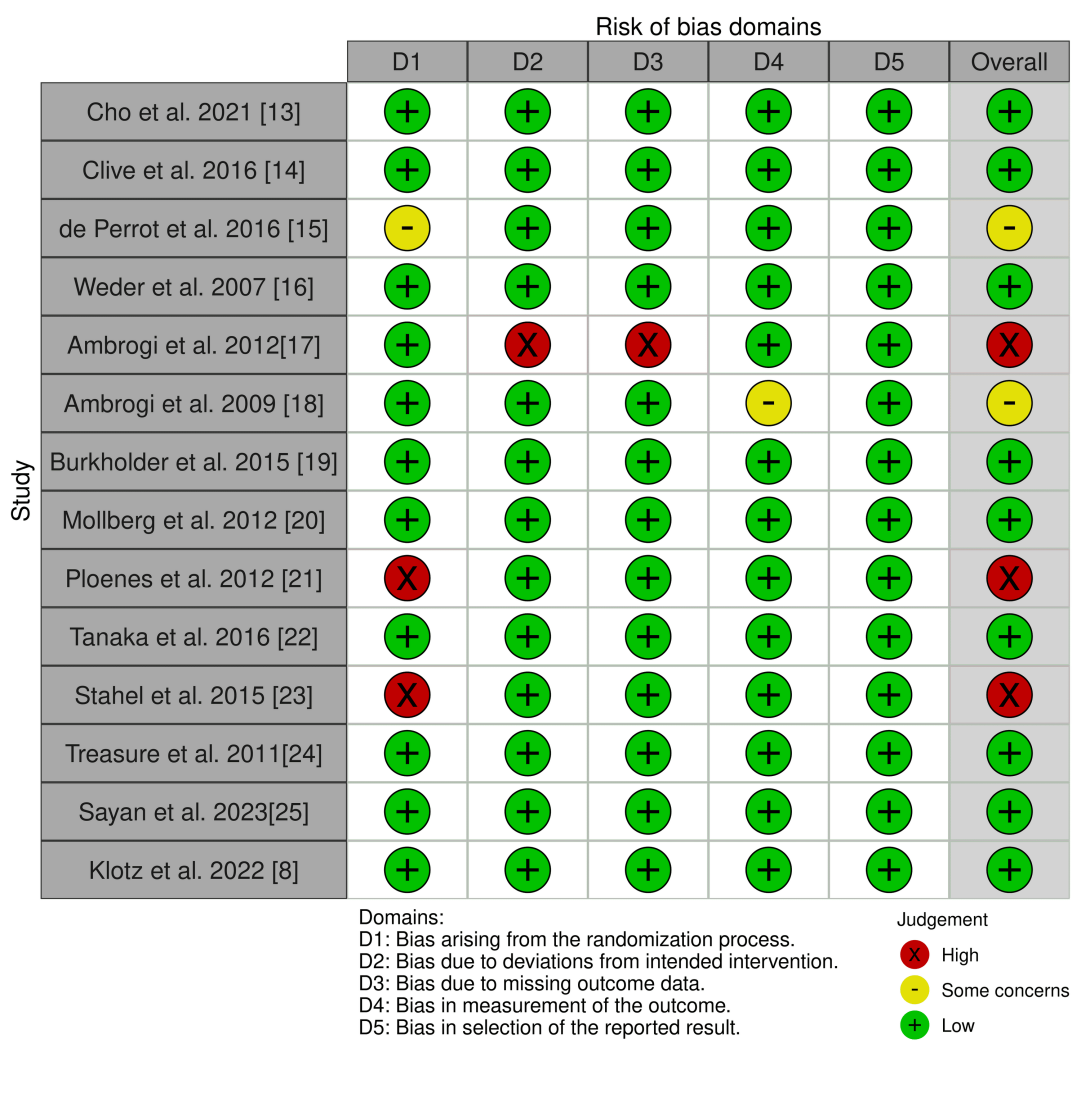
Traffic light plot of risk of bias in the included studies

As mentioned earlier, RoB version 2 was used to assess the risk for all the primary studies selected for meta-analysis. We used the Cochrane RoB tool to create a “traffic lights” plot for the final assessment (Figure 2).

Meta-analysis

The meta-analysis was conducted using Review Manager (RevMan) software to analyze both dichotomous and continuous data. Dichotomous variables, such as complications, were assessed using ORs with 95% CIs, while continuous variables, including lethargy, physical function, and social function, were evaluated using MDs or SMDs depending on data consistency. Complications were analyzed to determine the risk associated with multimodal surgical resection and targeted therapy. Lethargy, physical function, and social function, which are important indicators of patient quality of life (QoL), were included as continuous variables to assess the impact of interventions on these domains.

The results of the analysis for each variable are visually represented in forest plots. These plots display the effect size, direction of association, and variability across included studies. For complications, the forest plot highlights the overall risk, with studies clustered around the pooled estimate. The plots for physical and social function illustrate the extent of improvement in these aspects of QoL following treatment, with most studies showing significant positive effects. Lethargy data demonstrate mixed outcomes, emphasizing the need for targeted strategies to address this symptom. The visual representation in forest plots aids in summarizing and interpreting the aggregated results effectively.

Complications

**Figure 3:**
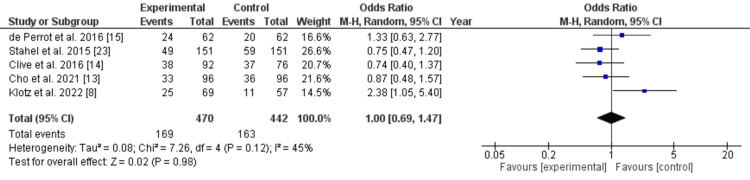
Forest plot for grade 3 complications in the included studies

The forest plot depicts a meta-analysis comparing complications between experimental and control groups in patients with early-stage MPM (Figure 3).

Data from five studies were included, each showing OR with 95% CIs. The pooled OR was 1.00 [95% CI: 0.69, 1.47], indicating no significant difference between the two groups. Individual study results varied, with ORs ranging from 0.74 to 2.38, but all CIs crossed the line of no effect (OR=1), showing no statistical significance in any study. The heterogeneity analysis revealed a Chi² of 7.26 (P=0.12) and an I² of 45%, suggesting moderate variability among studies. The diamond representing the pooled estimate lay centered at OR=1, confirming no overall difference in complications. The lack of significant findings highlights the need for more consistent protocols and larger sample sizes in future studies. Moderate heterogeneity implies differences in study populations or methodologies that may influence outcomes. Overall, the analysis suggested that experimental treatments do not increase or decrease the risk of complications compared to controls.

Lethargy

**Figure 4:**
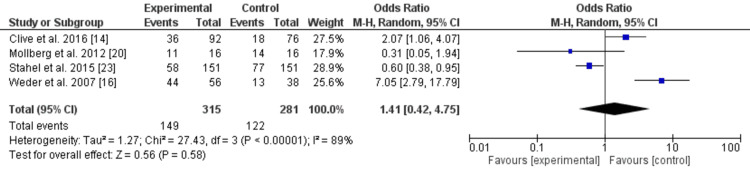
Forest plot for lethargy in the included studies

The forest plot given below (Figure 4) displays a meta-analysis of lethargy rates between the experimental and control groups, incorporating data from four studies.

The pooled OR was 1.39 (95% CI: 0.42, 4.52), suggesting no statistically significant difference between the groups. Individual studies showed varying ORs, ranging from 0.31 to 6.49, with the CIs of three studies crossing the line of no effect (OR=1), indicating no statistical significance. However, one study (Weder et al., 2007) showed a higher OR (6.49) with a wide CI, implying potential variability.

Heterogeneity was substantial, with a Chi² of 25.87 (P<0.0001) and an I² of 88%, indicating high variability among studies. This level of heterogeneity suggests that differences in study designs, populations, or definitions of lethargy may impact the pooled results. The diamond representing the pooled estimate crosses the line of no effect, reaffirming the lack of significant findings. 

Physical Function

**Figure 5:**
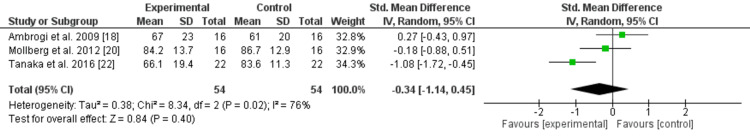
Forest plot for physical function in the included studies

The forest plot, involving three studies (Figure 5), compared the impact of experimental and control interventions on physical function in patients with MPM.

The pooled SMD was -0.34 (-1.14, 0.45), indicating a non-significant trend favoring the control group, as the CI crosses zero. Heterogeneity was substantial (I² =76%, P=0.02), suggesting variability in study results. The test for overall effect yielded a Z=0.84, P=0.40, confirming that there was no statistically significant difference in physical function outcomes between the experimental and control groups across the studies. 

Social Function

**Figure 6:**
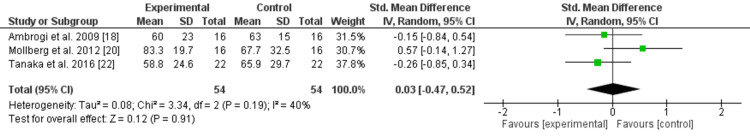
Forest plot for social function in the included studies

The forest plot for social function (Figure 6) illustrates a meta-analysis of three studies, with a pooled SMD of 0.01 (95% CI: -0.52, 0.53), indicating no statistically significant difference between the experimental and control groups.

Individual SMDs ranged from -0.33 to 0.57, and all CIs included the line of no effect (SMD=0). Heterogeneity was moderate, as evidenced by a Chi² of 3.79 (P=0.15) and an I² of 47%, suggesting some variability across the studies. 

Discussion

This systematic review and meta-analysis included a total of 14 studies, providing comprehensive insights into the effects of multimodal surgical resection and targeted therapies for early-stage MPM. The meta-analysis revealed mixed findings. For complications, there was no statistically significant difference between the experimental and control groups. Similarly, variables such as lethargy, physical function, and social function did not show clinically significant improvements, although some studies indicated trends favoring experimental approaches. These findings are generally consistent with Schwartz et al, who reported that although QoL remained compromised six months postoperatively, patients undergoing P/D experienced better physical and social functioning compared to those undergoing EPP [26]. In contrast, Abdel-Rahman et al. found no overall QoL benefit with EPP-based multimodal therapy over standard therapy and highlighted a higher rate of serious adverse events with EPP, further reinforcing the caution required when interpreting QoL outcomes across modalities [27].

EPP following radiation therapy has demonstrated positive short- and long-term outcomes, although its implementation is technically challenging due to substantial risks, including complications [13,15,23,24]. These complications may influence survival outcomes beyond the immediate postoperative period. Similarly, Abdel-Rahman et al. highlighted the high morbidity associated with EPP, noting a higher number of serious adverse events compared to non-radical therapy, suggesting that these risks may offset potential survival benefits [27].

Furthermore, the evidence does not support the routine use of prophylactic radiation therapy in all mesothelioma patients undergoing large-bore thoracic procedures [13]. The preferred treatment approach for resectable MPM involves accelerated hemithoracic intensity-modulated radiotherapy (IMRT) followed by EPP [14]. This strategy has shown encouraging operability rates and survival outcomes, particularly in patients with the epithelial subtype. Patients undergoing EPP demonstrated a median survival of 23 months, aligning with results from single-center studies. Importantly, this strategy was not associated with an increase in psychological distress, suggesting a balance between aggressive treatment and preserving patient well-being [15-17]. In contrast, Abdel-Rahman et al. reported median overall survival of 14.4 months for EPP patients, lower than standard therapy (19.5 months), underscoring variability in outcomes depending on institutional expertise and patient selection [27]. EPP significantly impacts QoL by alleviating symptoms and improving mental and physical health domains, particularly in symptomatic patients. The postoperative Short-Form-36 physical component score was a strong prognostic indicator, alongside biological factors. In contrast, extended P/D preserved pulmonary function but had mixed impacts on health-related quality of life (HRQoL). For patients with minimal symptoms, EPD did not improve HRQoL but provided substantial relief for those with baseline symptoms [18-22]. This aligns with Schwartz et al., who reported better preserved lung function (forced expiratory volume in one second or FEV1 and forced vital capacity or FVC) and improved QoL domains (global health, physical function) in P/D patients compared to EPP, though they also noted that pain and cough scores were similar between the two [26].

Radical P/D was associated with declines in physical function and exercise capacity post-surgery, although lung function was maintained [19-21]. These findings highlight the need to tailor surgical approaches to individual patient profiles, balancing symptom relief and functional preservation. This is echoed by Abdel-Rahman et al. (2018), whose Cochrane review found no survival benefit and increased toxicity associated with adding high-dose hemithoracic radiotherapy to EPP, suggesting limited justification for its routine use in multimodal settings [27].

Importantly, hemithoracic radiotherapy following EPP and neoadjuvant chemotherapy is not routinely recommended due to its limited benefits and high morbidity [23]. While EPP within a trimodal therapy framework may not universally improve survival or HRQoL and carries significant risks, lung-sparing techniques such as extended P/D combined with hyperthermic intrathoracic chemoperfusion (HITOC) appear safer and more effective. This approach is particularly favorable for localized epithelioid pleural mesothelioma, offering a practical and safe alternative to trimodal EPP with potential for improved overall survival. These findings underscore the critical role of individualized, multimodal treatment strategies in optimizing outcomes for patients with MPM [8,25]. The findings from Schwartz et al. (2018) further support the preferential use of P/D over EPP, particularly in patients where preserving lung function and achieving better QoL outcomes is a priority [26].

This study has several limitations that should be acknowledged. First, the included studies exhibited moderate to high heterogeneity in terms of patient populations, treatment protocols, and outcome measures, which may impact the generalizability of the findings. Second, most studies included in the meta-analysis were observational or retrospective in design, introducing the potential for selection bias and confounding factors. Third, sample sizes were relatively small in several studies, reducing the statistical power to detect significant differences, especially for subgroup analyses. Furthermore, variations in the definitions of complications and QoL metrics across studies made direct comparisons challenging. The lack of standardized reporting on baseline patient characteristics, such as performance status and comorbidities, may have influenced outcome interpretation. Additionally, the follow-up durations varied significantly between studies, limiting the ability to comprehensively assess long-term survival and QoL outcomes. Finally, publication bias cannot be excluded, as studies reporting positive outcomes are more likely to be published. Moreover, the absence of large-scale randomized controlled trials (RCTs) limits the conclusions. These limitations highlight the need for well-designed, multicentre RCTs with standardized protocols to better evaluate the effectiveness and safety of multimodal treatment strategies for MPM.

## Conclusions

This systematic review and meta-analysis highlights the complexities of MPM and underscores the importance of multimodal surgical resection and personalized targeted therapy for individual patient profiles. While EPP can alleviate symptoms and improve certain aspects of QoL, it is associated with significant risks, particularly for complications and declines in pulmonary function. In contrast, lung-sparing approaches like extended P/D offer better preservation of pulmonary function and may provide superior QoL outcomes, especially for patients with baseline symptoms.
